# Combined biofeedback and vestibular rehabilitation therapy for vestibular migraine: clinical efficacy and neurobiochemical correlates

**DOI:** 10.3389/fneur.2026.1861115

**Published:** 2026-06-23

**Authors:** Tengteng Zhang, Ying Zhang, Jinghui Sun, Luting Lv, Hongwei Wang, He Zhang, Jie Li, Jiandong Wang, Zhidan Yu, Shuqin Wu

**Affiliations:** 1 Department of Neurology, The Second Affiliated Hospital of Qiqihar Medical University, Qiqihar, Heilongjiang, China; 2 Ruby Street Community Health Service, The First Affiliated Hospital of Qiqihar Medical University, Qiqihar, Heilongjiang, China; 3 College of Pathology, Qiqihar Medical University, Qiqihar, Heilongjiang, China

**Keywords:** biofeedback therapy, calcitonin gene-related peptide, neurotransmitters, vestibular migraine, vestibular rehabilitation

## Abstract

**Background:**

Vestibular migraine (VM) is a prevalent cause of recurrent vertigo with limited standardized treatment options. While vestibular rehabilitation and biofeedback therapy have individually demonstrated efficacy, their combined application and effects on neurobiochemical markers remain unexplored.

**Methods:**

This prospective comparative study enrolled 148 patients with VM at a single tertiary center between January 2022 and March 2024. Patients were allocated to four groups (*n* = 37 each): routine intervention (Group A), vestibular rehabilitation (Group B), biofeedback therapy (Group C), or combined biofeedback and vestibular rehabilitation (Group D). Treatment efficacy, psychological outcomes (Hospital Anxiety and Depression Scale), vertigo disability (Dizziness Handicap Inventory), balance function (Berg Balance Scale), vestibular function parameters, cerebral blood flow velocities, and serum biomarkers (5-hydroxytryptamine, calcitonin gene-related peptide, *γ*-aminobutyric acid, and acetylcholine) were assessed at baseline and after 4 weeks.

**Results:**

Combined therapy achieved a significantly higher total effective rate (94.59%) compared to biofeedback (78.38%), vestibular rehabilitation (75.68%), and routine intervention (67.57%) (*p* = 0.035). The total effective rate was defined *a priori* as the proportion of patients whose headache, vertigo, and associated symptoms either resolved completely (markedly effective) or improved substantially (effective) relative to baseline. Group D demonstrated superior improvements in anxiety and depression scores, vertigo disability, and balance function compared to all other groups (all *p* < 0.001). Vestibular function parameters, including spontaneous nystagmus velocity, canal paresis, and directional preponderance, improved most substantially with combined therapy. Serum 5-hydroxytryptamine and *γ*-aminobutyric acid levels increased significantly, while calcitonin gene-related peptide and acetylcholine levels decreased, with the greatest modulation observed in the combined therapy group.

**Conclusion:**

Combined biofeedback and vestibular rehabilitation therapy provides superior clinical efficacy and favorable neurobiochemical modulation in patients with vestibular migraine, supporting its application as a promising non-pharmacological treatment strategy.

## Introduction

Vestibular migraine (VM) is a chronic neurological disorder characterized by recurrent episodes of vertigo and dizziness, frequently accompanied by or alternating with migraine headaches (1). Recent epidemiological surveys indicate that VM accounts for more than 10% of patients presenting to vertigo outpatient clinics, establishing it as one of the most common causes of both acute and chronic dizziness (2). Despite its growing clinical significance, the pathophysiological mechanisms underlying VM remain incompletely understood (3, 4).

Current evidence suggests that VM results from integrated dysfunction within sensory processing networks, involving complex interactions among abnormal central vestibular signal processing, neurotransmitter imbalance, trigeminal vascular system activation, and cortical spreading depression (5). Several neurobiochemical markers have been implicated in VM pathophysiology. Serotonin (5-hydroxytryptamine, 5-HT) plays a critical role in vestibular function regulation, and its imbalance may destabilize the vestibular system (6). Calcitonin gene-related peptide (CGRP), a vasodilatory neuropeptide released primarily by trigeminal ganglion neurons, has been shown to drive headache symptoms and directly induce vertigo by affecting vestibular nucleus excitability (7). Additionally, the balance between *γ*-aminobutyric acid (GABA), the principal inhibitory neurotransmitter, and acetylcholine (ACh), an important excitatory neurotransmitter, is essential for maintaining central vestibular stability (8–10).

Beyond these neurochemical features, VM is increasingly understood as a multidimensional disorder. Anxiety and depression are among its most frequent comorbidities and share overlapping serotonergic and limbic mechanisms with migraine, forming a bidirectional relationship in which psychological distress both intensifies and is intensified by recurrent vertigo (11). At the same time, dysfunction is not confined to a single peripheral site; rather, VM reflects altered central vestibular processing that manifests across multiple objective measures of vestibular compensation rather than as one characteristic abnormality (5). The trigemino-vascular activation that drives the headache component is also accompanied by changes in cerebrovascular tone, linking the disorder to measurable cerebral hemodynamic alterations (7). Taken together, these psychological, central-vestibular, and cerebrovascular dimensions, alongside the neurochemical changes described above, define the domains through which the burden of VM is expressed and through which a successful therapy would be expected to act.

Clinical management of VM remains challenging, as no unified standardized treatment protocol currently exists. Although pharmacological therapy provides benefit for some patients, others respond poorly or cannot tolerate medication side effects. Consequently, non-pharmacological treatment strategies have gained increasing attention (12). Vestibular rehabilitation has demonstrated efficacy in relieving dizziness and discomfort through customized exercises designed to improve sensory integration and promote vestibular compensation (13, 14). Biofeedback therapy converts physiological signals into visual or auditory feedback, enabling patients to learn conscious regulation of their autonomic nervous function (14, 15). Consistent with this growing interest, a recent prospective trial of mindfulness-based stress reduction demonstrated meaningful reductions in dizziness handicap and improvements in quality of life among patients with VM, further underscoring the therapeutic potential of interventions that target the autonomic and psychological dimensions of the disorder (16).

Theoretically, combined application of biofeedback and vestibular rehabilitation may produce synergistic effects by addressing complementary aspects of VM pathophysiology. Biofeedback may optimize autonomic nervous system regulation and reduce central sensitization, potentially enhancing patient tolerance and responsiveness to vestibular rehabilitation training (17, 18). However, clinical evidence supporting this combined approach remains limited, and its effects on neurobiochemical markers have not been systematically evaluated.

Therefore, the present study aimed to investigate the efficacy of biofeedback combined with vestibular rehabilitation in patients with VM. Specifically, we sought to evaluate treatment effects on clinical outcomes including anxiety, depression, vertigo disability, balance function, and vestibular function parameters, while simultaneously examining changes in serum levels of 5-HT, CGRP, GABA, and ACh to elucidate potential underlying mechanisms. Our findings may provide evidence-based support for clinical application of this combined therapeutic approach and offer new insights for precision treatment of VM.

## Methods

### Study design

**Figure 1:**
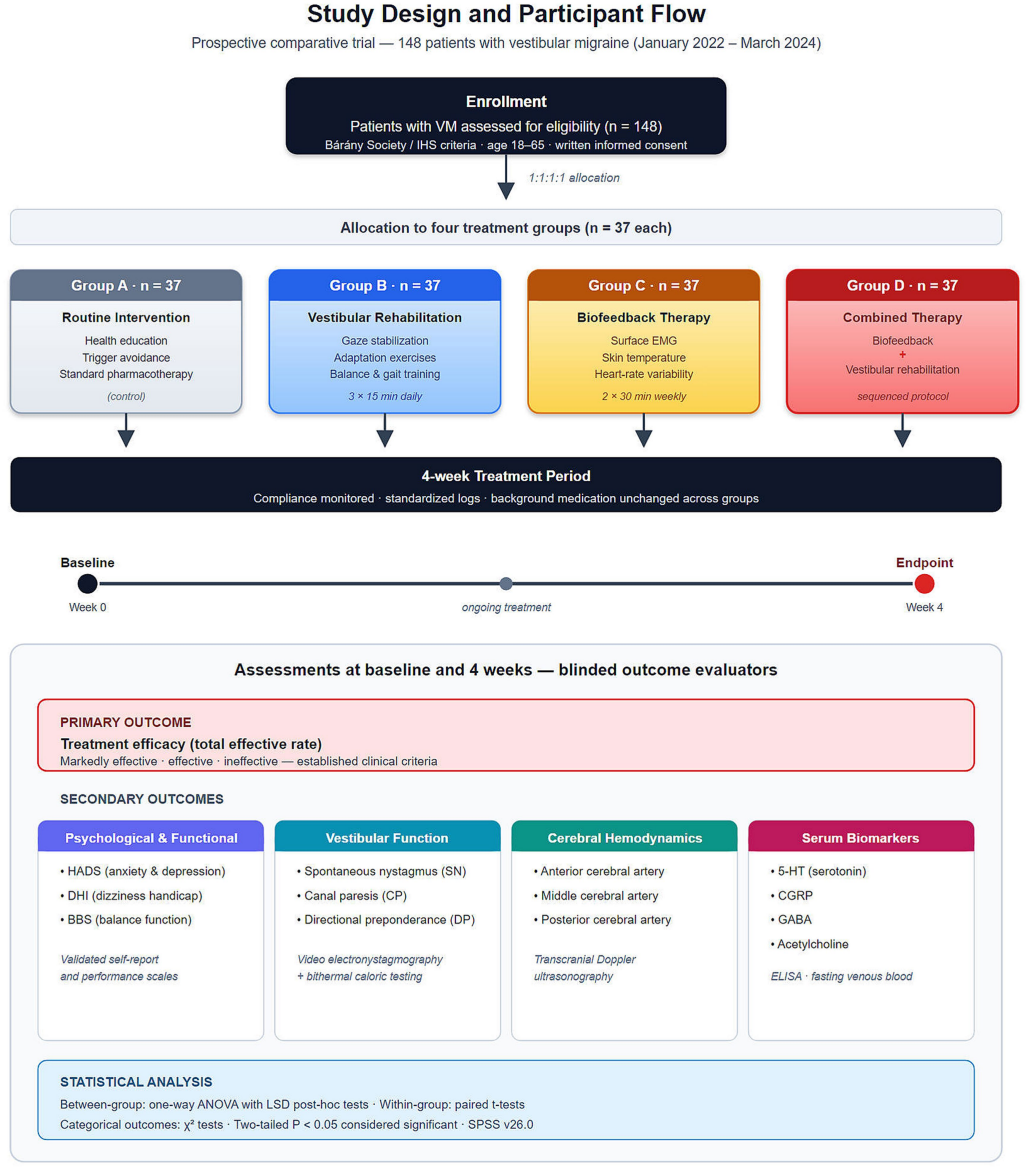
Study design and participant flow. Schematic overview of the prospective comparative trial. A total of 148 patients with vestibular migraine (VM) were allocated 1:1:1:1 to Group A (routine intervention), Group B (vestibular rehabilitation therapy), Group C (biofeedback therapy), or Group D (combined biofeedback and vestibular rehabilitation therapy), with 37 patients per group. All patients received their assigned intervention over 4 weeks. Assessments were performed at baseline and at 4 weeks by personnel blinded to treatment allocation. The primary outcome was treatment efficacy (total effective rate); secondary outcomes included psychological and functional scales (HADS, DHI, BBS), video electronystagmography parameters (SN, CP, DP), transcranial Doppler cerebral blood flow velocities (ACA, MCA, PCA), and ELISA-measured serum biomarkers (5-HT, CGRP, GABA, ACh).

This prospective, non-randomized comparative study evaluated patients with VM treated at the Second Affiliated Hospital of Qiqihar Medical University between January 2022 and March 2024. Patients were allocated to four treatment groups based on clinical presentation and patient preference: Group A (routine intervention), Group B (vestibular rehabilitation therapy), Group C (biofeedback therapy), and Group D (biofeedback combined with vestibular rehabilitation therapy). Allocation followed a 1:1:1:1 distribution ratio. The overall study design, intervention protocols, and outcome assessment framework are summarized in Figure 1.

This study was approved by the Ethics Committee of the Second Affiliated Hospital of Qiqihar Medical University. All patients provided written informed consent prior to participation. The study was conducted in accordance with the Declaration of Helsinki and its subsequent amendments.

### Participants

#### Eligibility criteria

Patients were eligible for inclusion if they met the following criteria:

Diagnosis of VM according to the diagnostic criteria established in the “Expert Consensus on Diagnosis and Treatment of Vestibular Migraine (19)” (The Chinese expert consensus used for diagnosis was developed from, and is consistent with, the criteria of the Bárány Society (20), and the International Headache Society, so the study population corresponds to internationally recognized definitions of vestibular migraine)

- Age between 18 and 65 years (the 18-to-65-year age range was applied to limit confounding from age-related conditions, such as presbyvestibulopathy and cerebral small-vessel disease, that could independently affect vestibular function, balance, and cerebral hemodynamics)
- Voluntary provision of written informed consent
- Availability of complete clinical data

Patients were excluded if they met any of the following criteria:

- Presence of other primary or secondary headache disorders
- Documented vestibular peripheral or central diseases, including benign paroxysmal positional vertigo, Menière’s disease, or vestibular neuritis
- Recent use of immunosuppressants, corticosteroids, vestibular suppressants, benzodiazepines, or other medications potentially affecting vestibular compensation or neurotransmitter levels, with “recent” defined as any use within the 4 weeks immediately preceding enrollment
- Diagnosis of severe malignant tumors or hematological or immunological disorders
- Severe psychiatric conditions, including major depressive disorder or schizophrenia requiring ongoing psychotropic pharmacotherapy, or a baseline Hospital Anxiety and Depression Scale depression subscale score greater than 15; milder anxiety or depressive symptoms, which are intrinsic to VM, were not grounds for exclusion
- Significant cardiac, hepatic, or renal insufficiency
- Pregnancy or lactation

### Group allocation and sample size

Treatment allocation was performed sequentially based on patient preference and clinical suitability, maintaining a 1:1:1:1 distribution across groups. This pragmatic allocation approach reflected real-world clinical decision-making while ensuring balanced group sizes. A total of 148 patients met the eligibility criteria, with 37 patients assigned to each of the four treatment groups. Baseline characteristics were compared across groups to ensure comparability prior to analysis (Table 1).

**Table 1:** Baseline demographic and clinical characteristics of study participants.

Characteristic	Group A (*n* = 37)	Group B (*n* = 37)	Group C (*n* = 37)	Group D (*n* = 37)	*F*/*χ*^2^	*p*-value
Sex, *n* (%)					0.547	0.908
Male	18 (48.65)	17 (45.95)	16 (43.24)	15 (40.54)		
Female	19 (51.35)	20 (54.05)	21 (56.76)	22 (59.46)		
Age, years	47.95 ± 5.10	48.77 ± 4.43	48.89 ± 4.60	49.35 ± 4.53	0.576	0.632
Body mass index, kg/m^2^	22.98 ± 1.25	23.01 ± 1.13	23.16 ± 1.08	23.18 ± 1.12	0.293	0.830
Disease duration, years	2.45 ± 0.77	2.72 ± 0.68	2.76 ± 0.70	2.85 ± 0.66	2.214	0.089
Family history of vertigo, *n* (%)	6 (16.22)	8 (21.62)	9 (24.32)	10 (27.03)	1.595	0.661

Data are presented as mean ± SD or *n* (%). Group A = routine intervention; Group B = vestibular rehabilitation; Group C = biofeedback therapy; Group D = combined therapy.

With 37 patients per group, this study provided approximately 80% power to detect a medium effect size (Cohen’s *d* = 0.6) between the combined therapy group and individual therapy groups at a two-sided significance level of 0.05.

### Interventions

All interventions were administered over a 4-week treatment period. Treatment compliance was monitored through attendance records and standardized treatment logs maintained by therapists.

#### Group A: routine intervention

Patients in Group A received standard care consisting of health education regarding lifestyle modifications, trigger avoidance, and pharmacological management without systematic vestibular rehabilitation or biofeedback training. Standard pharmacological management included prophylactic and abortive medications as clinically indicated, which were maintained consistently across all groups to isolate the effects of the rehabilitation interventions. Prophylactic agents were drawn from the classes most commonly used for VM at our center and comprised flunarizine (5 to 10 mg at night), beta-blockers (metoprolol 25 to 50 mg daily or propranolol 20 to 40 mg daily), the tricyclic agent amitriptyline (12.5 to 25 mg at night), and topiramate (25 to 50 mg daily), prescribed singly and titrated to tolerance. Abortive treatment of acute attacks consisted of vestibular symptom relief with betahistine and, when required, short-acting antiemetics, together with simple analgesics or triptans for accompanying headache. To preserve comparability, each patient’s background regimen was held constant in class and dose for the 4-week study period across all four groups, so that any between-group differences could be attributed to the rehabilitation interventions rather than to changes in pharmacotherapy.

#### Group B: vestibular rehabilitation therapy

Vestibular rehabilitation therapy was administered three times daily, with each session lasting 15 min. The protocol comprised three progressive components. First, gaze stabilization exercises required patients to maintain visual fixation on a stationary target while performing progressively faster horizontal and vertical head movements, alternating between eyes-open and eyes-closed conditions. Second, adaptation exercises involved rapid sagittal head nodding movements while sustaining gaze on a target following a 45-degree head rotation to the left or right. Third, balance and gait training consisted of walking forward 10 to 15 steps while simultaneously performing multi-axis head movements, with alternating eyes-open and eyes-closed conditions. Exercise intensity was progressively increased based on patient tolerance, and home exercise compliance was reinforced through weekly therapist supervision. The program was prescribed, taught, and supervised by two licensed physical therapists with formal training in vestibular rehabilitation, each with more than 5 years of clinical experience in the management of dizziness and balance disorders. The therapists conducted the initial instruction and weekly supervised sessions, progressed exercise difficulty according to standardized criteria, and verified the accuracy of the home program; they were not involved in outcome assessment, which was performed independently by separate blinded evaluators.

#### Group C: biofeedback therapy

Biofeedback therapy was delivered using the BF06 Intelligent Biofeedback Therapeutic Instrument (China Institute of Aeronautical Medicine) twice weekly, with each session lasting 30 min. Patients were instructed to abstain from caffeine, alcohol, and tea for at least 24 h prior to each session. Treatment was conducted in a quiet, comfortable environment with subdued lighting.

Each session commenced with a 5-min preparatory phase incorporating deep diaphragmatic breathing and progressive muscle relaxation techniques. Surface electromyography (EMG) electrodes were positioned on the frontalis muscle or bilateral trapezius muscle bundles based on the patient’s predominant somatic symptoms. Patients received real-time visual and auditory feedback to facilitate recognition and voluntary reduction of EMG activity. Concurrently, a skin temperature sensor was attached to the fingertip of the index finger, and patients were trained to increase peripheral temperature through cognitive techniques to induce a relaxation response. Heart rate variability biofeedback was incorporated to synchronize respiratory rhythm with cardiac oscillations, optimizing autonomic balance at individualized respiratory rates. Baseline values for EMG activity, skin temperature, and heart rate variability were recorded during the initial session and served as reference thresholds for subsequent treatments.

#### Group D: combined biofeedback and vestibular rehabilitation therapy

Patients in Group D received both biofeedback therapy and vestibular rehabilitation therapy according to the protocols described for Groups B and C, respectively. Biofeedback sessions were consistently scheduled to precede vestibular rehabilitation sessions to optimize autonomic nervous system regulation and reduce sympathetic overactivity prior to sensorimotor training, thereby enhancing patient tolerance and training efficacy.

### Outcome measures

#### Primary outcome: treatment efficacy

Treatment efficacy was evaluated after 4 weeks according to established clinical criteria. To reduce reliance on a purely global impression, response was graded against the change in vertigo burden between baseline and 4 weeks, anchored to a quantitative threshold and corroborated by the change in Dizziness Handicap Inventory (DHI) score. A composite vertigo index incorporating attack frequency, duration, and severity was recorded at both time points, and the percentage reduction was calculated for each patient. Operationally, this index was calculated for each patient as the product of three dimensions documented over the 7 days preceding each assessment: the number of attacks during that week (frequency), the mean duration of an individual attack in minutes (duration), and the mean peak intensity of an attack rated on a 0-to-10 visual analog scale (severity). Because the three components were multiplied, a reduction in any one of them lowered the index, and the percentage change from baseline to 4 weeks expressed the overall change in vertigo burden in a single value. For instance, a patient reporting 3 severe attacks during the index week, each lasting on average 5 min with a mean peak intensity of 8 on the 0-to-10 scale, would have a baseline index of 3 × 5 × 8 = 120; if, following treatment, the same patient reported a single attack lasting 2 min at an intensity of 4, the index would fall to 1 × 2 × 4 = 8, corresponding to a reduction of approximately 93% and thereby meeting the 80% threshold required for a markedly effective response. A response was classified as “markedly effective” when headache, vertigo, and associated symptoms resolved completely or the vertigo index decreased by at least 80%, accompanied by a DHI reduction of at least 30 points; “effective” when the vertigo index decreased by 30 to 79% with a DHI reduction of at least 18 points, the established minimal clinically important difference for that instrument, corresponding to the smallest change that exceeds the 95% confidence interval for measurement error and therefore reflects a true change rather than test–retest variability (21); and “ineffective” when the vertigo index decreased by less than 30% and the DHI improvement did not reach 18 points. The total effective rate was defined *a priori* as the proportion of patients meeting the markedly effective or effective thresholds. Anchoring the categories to a predefined percentage change and to a validated patient-reported instrument was intended to make the distinction between substantial and non-substantial improvement explicit and reproducible rather than dependent on unaided clinical judgment.

### Secondary outcomes

#### Psychological assessment

Anxiety and depression were assessed using the Hospital Anxiety and Depression Scale (HADS), which comprises two subscales (anxiety and depression), each scored from 0 to 21 points, yielding a total score ranging from 0 to 42 points. Higher scores indicate greater symptom severity.

#### Vertigo assessment

The Dizziness Handicap Inventory (DHI) was used to evaluate the functional, emotional, and physical impact of vertigo on daily activities. Total scores range from 0 to 100 points, with higher scores indicating greater disability.

#### Balance function

Balance function was assessed using the Berg Balance Scale (BBS), which evaluates performance across 14 functional tasks including sitting, standing, and dynamic balance activities. Total scores range from 0 to 56 points, with higher scores indicating better balance.

#### Vestibular function parameters

Vestibular function was evaluated using video electronystagmography (Interacoustics, Denmark). Parameters assessed included spontaneous nystagmus slow-phase velocity (SN, °/s), canal paresis value (CP, %), and directional preponderance (DP, %). Caloric testing was performed using standard bithermal stimulation protocols, with canal paresis calculated using the Jongkees formula.

#### Cerebral blood flow

Transcranial Doppler ultrasonography was performed using a digital color Doppler diagnostic system (Apogee 8G, Shantou Ultrasonic Instrument Research Institute Co., Ltd.) to measure mean blood flow velocity in bilateral anterior cerebral arteries (ACA), middle cerebral arteries (MCA), and posterior cerebral arteries (PCA). All measurements were obtained by the same experienced sonographer who was blinded to group allocation, using standardized insonation protocols.

#### Serum biomarkers

Fasting venous blood samples (3 mL) were collected between 7:00 and 9:00 a.m. to minimize circadian variation and centrifuged at 3,000 rpm (radius: 8 cm) for 10 min to obtain serum. Serum samples were stored at −80 °C until analysis. Concentrations of 5-hydroxytryptamine (5-HT), calcitonin gene-related peptide (CGRP), *γ*-aminobutyric acid (GABA), and acetylcholine (ACh) were quantified using commercial enzyme-linked immunosorbent assay (ELISA) kits (Beckman Coulter Trading China Co., Ltd.) according to the manufacturer’s instructions. All samples were analyzed in duplicate, and the mean values were used for statistical analysis. Intra-assay and inter-assay coefficients of variation were <10% for all biomarkers.

#### Assessment timeline

All outcome measures were assessed at baseline (before treatment initiation) and after 4 weeks of treatment. Assessments were performed by trained personnel who were blinded to treatment allocation.

#### Statistical analysis

Statistical analyses were performed using SPSS version 26.0 (IBM Corporation, Armonk, NY, USA). The Shapiro–Wilk test was used to assess normality of continuous variables; all continuous variables demonstrated approximately normal distributions. Normally distributed continuous data are presented as mean ± standard deviation. Within-group comparisons (before vs. after treatment) were performed using paired *t*-tests. Between-group comparisons were conducted using one-way analysis of variance (ANOVA), with *post-hoc* pairwise comparisons performed using the least significant difference (LSD) test when ANOVA results were significant. Categorical variables are presented as frequencies and percentages and were compared using chi-square tests or chi-square tests for trend, as appropriate. A two-tailed *p*-value < 0.05 was considered statistically significant.

## Results

### Baseline characteristics

A total of 148 patients with VM were enrolled and allocated to four treatment groups, with 37 patients in each group. Baseline demographic and clinical characteristics were comparable across the four groups (Table 1). The mean age ranged from 47.95 ± 5.10 years in Group A to 49.35 ± 4.53 years in Group D (*F* = 0.576, *p* = 0.632). Female patients constituted the majority in all groups, ranging from 51.35% in Group A to 59.46% in Group D (*χ*^2^ = 0.547, *p* = 0.908). Mean body mass index was similar across groups, ranging from 22.98 ± 1.25 kg/m^2^ in Group A to 23.18 ± 1.12 kg/m^2^ in Group D (*F* = 0.293, *p* = 0.830). Disease duration ranged from 2.45 ± 0.77 years in Group A to 2.85 ± 0.66 years in Group D (*F* = 2.214, *p* = 0.089). The proportion of patients with a family history of vertigo ranged from 16.22% in Group A to 27.03% in Group D (*χ*^2^ = 1.595, *p* = 0.661). No statistically significant differences were observed in any baseline characteristic among the four groups, confirming the comparability of study populations.

### Treatment efficacy

The combined therapy demonstrated superior efficacy compared to individual or routine interventions (Table 2). After 4 weeks of treatment, Group D achieved a total effective rate of 94.59%, comprising 48.65% markedly effective and 45.95% effective responses, with only 5.41% of patients classified as non-responders. This efficacy rate was significantly higher than that observed in Group C (78.38%), Group B (75.68%), and Group A (67.57%) (*χ*^2^ = 8.610, *p* = 0.035). While both individual therapies (vestibular rehabilitation and biofeedback) demonstrated numerically higher response rates than routine intervention, the combined approach yielded the most pronounced clinical benefit.

**Table 2:** Treatment efficacy after 4 weeks of treatment.

Group	*n*	Markedly effective, *n* (%)	Effective, *n* (%)	Ineffective, *n* (%)	Total effective rate, *n* (%)
Group A	37	11 (29.73)	14 (37.84)	12 (32.43)	25 (67.57)
Group B	37	12 (32.43)	16 (43.24)	9 (24.32)	28 (75.68)
Group C	37	13 (35.14)	16 (43.24)	8 (21.62)	29 (78.38)
Group D	37	18 (48.65)	17 (45.95)	2 (5.41)	35 (94.59)ᵃᵇᶜ
*χ* ^2^					8.610
*p*-value					0.035

ᵃ*p* < 0.05 vs. Group A; ᵇ*p* < 0.05 vs. Group B; ᶜ*p* < 0.05 vs. Group C. Group A = routine intervention; Group B = vestibular rehabilitation; Group C = biofeedback therapy; Group D = combined therapy.

### Psychological outcomes and functional assessments

#### Anxiety and depression

All four treatment groups demonstrated significant reductions in HADS scores following the 4-week intervention period (Table 3). The magnitude of improvement varied substantially across groups. Group D exhibited the greatest reduction in psychological distress, with HADS scores decreasing from 25.45 ± 2.14 at baseline to 13.65 ± 1.92 after treatment (*p* < 0.05). This post-treatment score was significantly lower than those observed in Group C (16.88 ± 2.04), Group B (17.01 ± 1.99), and Group A (20.45 ± 1.86) (*F* = 74.769, *p* < 0.001). Both individual therapy groups achieved significantly greater reductions in anxiety and depression compared to routine intervention (all *p* < 0.05).

**Table 3:** Psychological outcomes, vertigo disability, and balance function before and after treatment.

Group	*n*	HADS score (points)		DHI score (points)		BBS score (points)	
		Baseline	4 weeks	Baseline	4 weeks	Baseline	4 weeks
Group A	37	24.88 ± 2.01	20.45 ± 1.86ᵈ	50.98 ± 7.10	27.88 ± 4.34ᵈ	18.25 ± 2.33	23.06 ± 3.10ᵈ
Group B	37	24.97 ± 2.08	17.01 ± 1.99ᵃᵈ	51.76 ± 6.44	22.02 ± 5.01ᵃᵈ	18.10 ± 2.28	27.98 ± 3.44ᵃᵈ
Group C	37	25.19 ± 2.10	16.88 ± 2.04ᵃᵈ	51.89 ± 6.85	21.87 ± 4.55ᵃᵈ	18.06 ± 2.31	28.11 ± 3.62ᵃᵈ
Group D	37	25.45 ± 2.14	13.65 ± 1.92ᵃᵇᶜᵈ	52.14 ± 6.77	16.44 ± 3.02ᵃᵇᶜᵈ	17.89 ± 2.23	35.71 ± 5.22ᵃᵇᶜᵈ
*F* value		0.551	74.769	0.201	43.806	0.155	65.315
*p*-value		0.648	<0.001	0.896	<0.001	0.926	<0.001

Data are presented as mean ± SD. HADS, Hospital Anxiety and Depression Scale; DHI, dizziness Handicap Inventory; BBS, Berg Balance Scale. ᵃ*p* < 0.05 vs. Group A; ᵇ*p* < 0.05 vs. Group B; ᶜ*p* < 0.05 vs. Group C; ᵈ*p* < 0.05 vs. baseline within the same group.

#### Vertigo disability

Parallel improvements were observed in vertigo-related disability as measured by the DHI (Table 3). Baseline DHI scores were comparable across groups, ranging from 50.98 ± 7.10 to 52.14 ± 6.77. Following treatment, Group D demonstrated the most substantial improvement, with scores decreasing to 16.44 ± 3.02 (*p* < 0.05). This post-treatment DHI score was significantly lower than those in Group C (21.87 ± 4.55), Group B (22.02 ± 5.01), and Group A (27.88 ± 4.34) (*F* = 43.806, *p* < 0.001). Groups B and C also showed significantly greater improvement compared to Group A (*p* < 0.05).

#### Balance function

Assessment of balance function using the BBS revealed significant improvements across all treatment groups, with the combined therapy group demonstrating superior outcomes (Table 3). From comparable baseline scores ranging from 17.89 ± 2.23 to 18.25 ± 2.33, Group D achieved the highest post-treatment BBS score of 35.71 ± 5.22, indicating marked enhancement of functional balance (*p* < 0.05). This improvement significantly exceeded those observed in Group C (28.11 ± 3.62), Group B (27.98 ± 3.44), and Group A (23.06 ± 3.10) (*F* = 65.315, *p* < 0.001). The individual therapy groups also demonstrated significantly greater balance improvement compared to routine intervention (all *p* < 0.05).

#### Vestibular function parameters

Objective vestibular function testing revealed significant improvements across all measured parameters following treatment (Table 4). Spontaneous nystagmus slow-phase velocity decreased substantially in all groups, with Group D achieving the lowest post-treatment value (0.41 ± 0.12 °/s), which was significantly lower than Group C (0.79 ± 0.20 °/s), Group B (0.82 ± 0.21 °/s), and Group A (1.57 ± 0.43 °/s) (*F* = 123.155, *p* < 0.001).

**Table 4:** Vestibular function parameters before and after treatment.

Group	*n*	SN (°/s)		CP (%)		DP (%)	
		Baseline	4 weeks	Baseline	4 weeks	Baseline	4 weeks
Group A	37	5.97 ± 1.86	1.57 ± 0.43ᵈ	71.55 ± 9.79	63.44 ± 8.87ᵈ	67.43 ± 9.94	48.86 ± 7.01ᵈ
Group B	37	6.13 ± 1.79	0.82 ± 0.21ᵃᵈ	71.78 ± 10.02	56.11 ± 9.04ᵃᵈ	67.66 ± 9.75	41.47 ± 6.55ᵃᵈ
Group C	37	6.17 ± 1.85	0.79 ± 0.20ᵃᵈ	71.97 ± 9.85	55.68 ± 8.71ᵃᵈ	67.87 ± 9.96	41.23 ± 6.67ᵃᵈ
Group D	37	6.22 ± 1.98	0.41 ± 0.12ᵃᵇᶜᵈ	72.13 ± 9.98	49.67 ± 7.78ᵃᵇᶜᵈ	68.09 ± 10.22	20.45 ± 4.25ᵃᵇᶜᵈ
*F*-value		0.124	123.155	0.024	15.847	0.030	143.096
*p*-value		0.946	<0.001	0.995	<0.001	0.993	<0.001

Data are presented as mean ± SD. SN, spontaneous nystagmus slow-phase velocity; CP, canal paresis; DP, directional preponderance. ᵃ*p* < 0.05 vs. Group A; ᵇ*p* < 0.05 vs. Group B; ᶜ*p* < 0.05 vs. Group C; ᵈ*p* < 0.05 vs. baseline within the same group.

**Figure 2:**
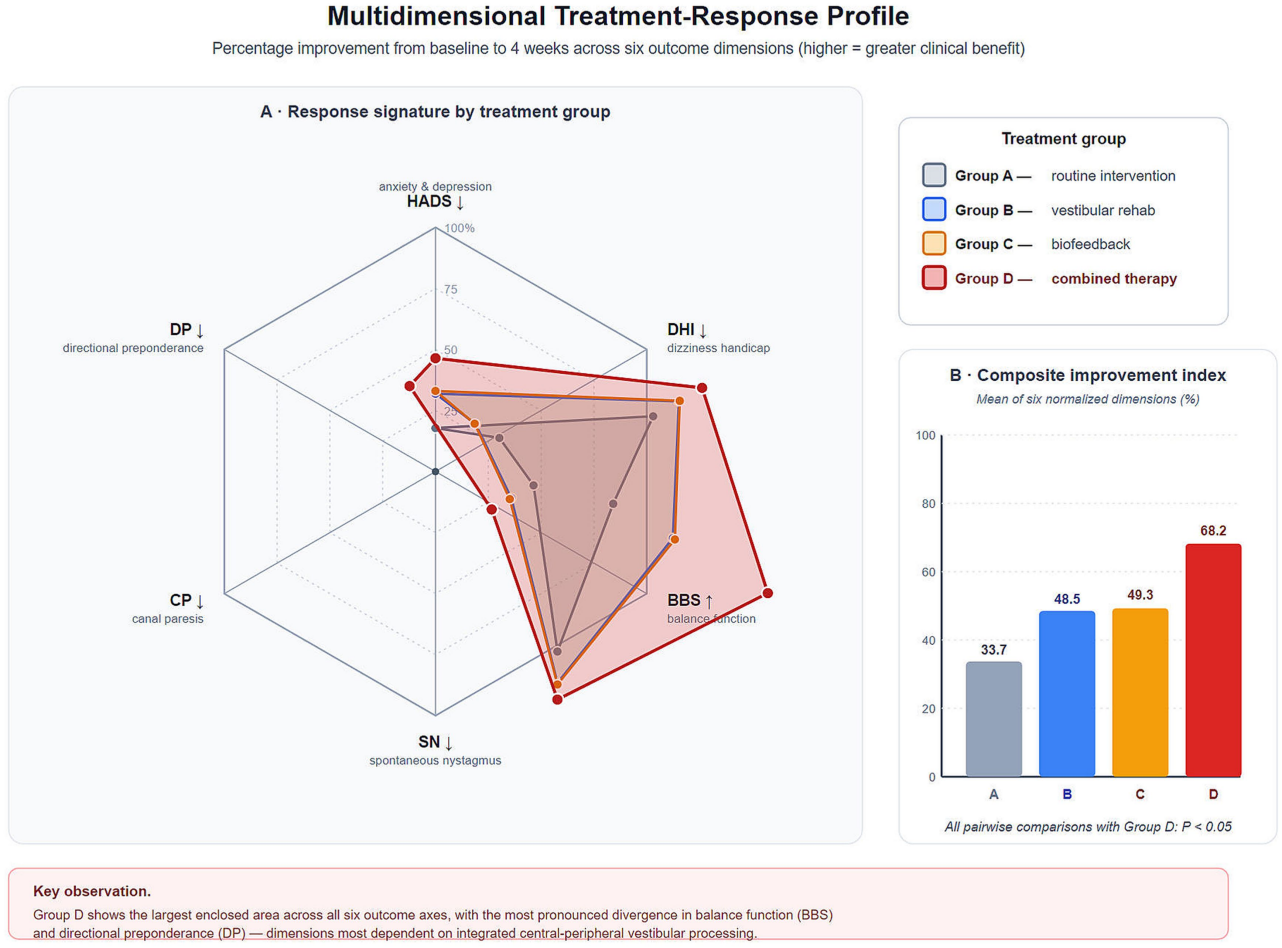
Multidimensional treatment-response profile. **(A)** Radar plot displaying the percentage improvement from baseline to 4 weeks across six outcome dimensions: HADS (Hospital Anxiety and Depression Scale), DHI (Dizziness Handicap Inventory), BBS (Berg Balance Scale), SN (spontaneous nystagmus slow-phase velocity), CP (canal paresis), and DP (directional preponderance). Each coloured polygon represents one treatment group; a larger enclosed area reflects greater multidimensional clinical benefit. Group D (combined therapy) produced the largest response signature, with the most pronounced divergence along the BBS and DP axes. **(B)** Composite improvement index, computed as the arithmetic mean of the six normalized improvement percentages, by group. All pairwise comparisons between Group D and Groups A, B, and C were statistically significant (*p* < 0.05).

Canal paresis values demonstrated similar patterns of improvement. Group D exhibited the greatest reduction, achieving a post-treatment C*p* value of 49.67 ± 7.78%, significantly lower than the values observed in Groups C (55.68 ± 8.71%), B (56.11 ± 9.04%), and A (63.44 ± 8.87%) (*F* = 15.847, *p* < 0.001). Directional preponderance showed the most dramatic improvements in the combined therapy group, with values decreasing from 68.09 ± 10.22% at baseline to 20.45 ± 4.25% after treatment in Group D. This reduction was significantly greater than those achieved in Groups C (41.23 ± 6.67%), B (41.47 ± 6.55%), and A (48.86 ± 7.01%) (*F* = 143.096, *p* < 0.001). Both individual therapy groups showed significantly greater improvement compared to Group A across all vestibular parameters (all *p* < 0.05). The composite multidimensional response profile across psychological, vertigo-disability, balance, and vestibular function outcomes is presented in Figure 2, illustrating that Group D consistently produced the largest percentage improvements and the highest composite improvement index across all six clinical dimensions.

#### Cerebral blood flow

Transcranial Doppler assessment revealed significant reductions in mean cerebral blood flow velocities across all arterial territories following treatment, suggesting normalization of previously elevated cerebrovascular hemodynamics (Table 5). In the anterior cerebral artery, mean blood flow velocity in Group D decreased from 69.12 ± 7.78 cm/s to 44.21 ± 6.12 cm/s (*p* < 0.05), significantly lower than post-treatment values in Groups C (47.98 ± 7.12 cm/s), B (48.13 ± 7.22 cm/s), and A (55.83 ± 8.02 cm/s) (*F* = 17.213, *p* < 0.001).

**Table 5:** Cerebral blood flow velocities before and after treatment.

Group	*n*	ACA (cm/s)		MCA (cm/s)		PCA (cm/s)	
		Baseline	4 weeks	Baseline	4 weeks	Baseline	4 weeks
Group A	37	67.94 ± 9.22	55.83 ± 8.02ᵈ	85.21 ± 10.09	71.12 ± 8.02ᵈ	56.94 ± 8.14	46.78 ± 6.23ᵈ
Group B	37	68.74 ± 8.11	48.13 ± 7.22ᵃᵈ	85.76 ± 10.44	64.08 ± 8.23ᵃᵈ	57.72 ± 7.43	40.05 ± 6.02ᵃᵈ
Group C	37	68.95 ± 8.02	47.98 ± 7.12ᵃᵈ	85.89 ± 10.94	63.66 ± 7.95ᵃᵈ	57.85 ± 7.22	39.91 ± 5.66ᵃᵈ
Group D	37	69.12 ± 7.78	44.21 ± 6.12ᵃᵇᶜᵈ	86.73 ± 11.23	57.43 ± 6.87ᵃᵇᶜᵈ	58.05 ± 8.85	35.43 ± 4.43ᵃᵇᶜᵈ
*F*-value		0.146	17.213	0.128	19.119	0.139	25.574
*p*-value		0.932	<0.001	0.943	<0.001	0.937	<0.001

Data are presented as mean ± SD. ACA, anterior cerebral artery; MCA, middle cerebral artery; PCA, posterior cerebral artery. ᵃ*p* < 0.05 vs. Group A; ᵇ*p* < 0.05 vs. Group B; ᶜ*p* < 0.05 vs. Group C; ᵈ*p* < 0.05 vs. baseline within the same group.

Middle cerebral artery blood flow velocity demonstrated comparable improvements, with Group D achieving the lowest post-treatment velocity of 57.43 ± 6.87 cm/s compared to Groups C (63.66 ± 7.95 cm/s), B (64.08 ± 8.23 cm/s), and A (71.12 ± 8.02 cm/s) (*F* = 19.119, *p* < 0.001). Posterior cerebral artery velocities followed the same hierarchical pattern, with Group D showing significantly lower values (35.43 ± 4.43 cm/s) than Groups C (39.91 ± 5.66 cm/s), B (40.05 ± 6.02 cm/s), and A (46.78 ± 6.23 cm/s) (*F* = 25.574, *p* < 0.001). Both individual therapy groups demonstrated significantly greater reductions in cerebral blood flow velocities compared to routine intervention across all three arterial territories (all *p* < 0.05).

#### Serum biomarker profiles

**Figure 3:**
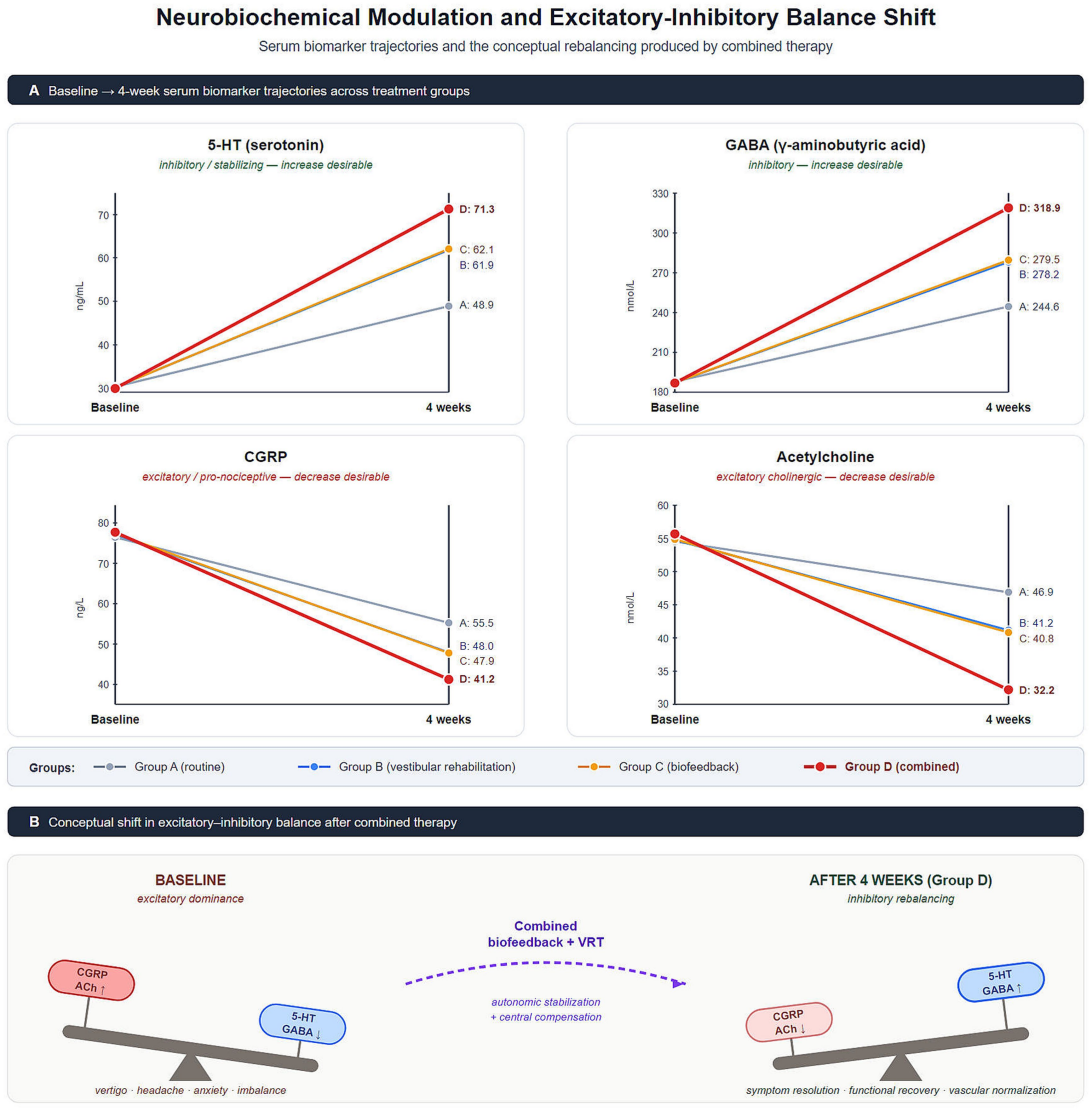
Neurobiochemical modulation and excitatory–inhibitory balance shift. **(A)** Slope graphs showing mean serum biomarker levels at baseline and after 4 weeks of treatment across all four groups. 5-HT (serotonin) and GABA (*γ*-aminobutyric acid) are inhibitory or stabilizing markers for which an increase indicates clinical benefit (upper panels); CGRP (calcitonin gene-related peptide) and acetylcholine (ACh) are excitatory markers for which a decrease indicates clinical benefit (lower panels). Group D (combined therapy) produced the largest shift in every biomarker. **(B)** Conceptual illustration of the excitatory–inhibitory neurotransmitter balance. At baseline, the excitatory arm (CGRP, ACh) predominates, corresponding clinically to vertigo, headache, anxiety, and imbalance. After 4 weeks of combined therapy in Group D, the balance shifts toward inhibitory predominance (increased 5-HT and GABA, decreased CGRP and ACh), paralleling symptom resolution, functional recovery, and cerebrovascular normalization.

Analysis of serum neurotransmitter and neuropeptide levels revealed distinct patterns of change following the intervention period, with the combined therapy group demonstrating the most favorable biomarker modulation (Table 6 and Figure 3). Group-wise baseline-to-4-week trajectories for each biomarker and the resulting shift in the excitatory–inhibitory balance are shown in Figure 3.

**Table 6:** Serum biomarker levels before and after treatment.

Group	*n*	5-HT (ng/mL)		CGRP (ng/L)		GABA (nmol/L)		ACh (nmol/L)	
		Baseline	4 weeks	Baseline	4 weeks	Baseline	4 weeks	Baseline	4 weeks
Group A	37	30.25 ± 3.41	48.89 ± 3.31ᵈ	76.94 ± 6.95	55.47 ± 6.19ᵈ	188.03 ± 26.09	244.55 ± 27.88ᵈ	54.52 ± 5.88	46.87 ± 4.43ᵈ
Group B	37	30.15 ± 3.39	61.89 ± 4.62ᵃᵈ	77.65 ± 7.43	48.01 ± 5.48ᵃᵈ	187.15 ± 25.44	278.23 ± 35.43ᵃᵈ	54.79 ± 6.13	41.15 ± 5.01ᵃᵈ
Group C	37	30.12 ± 3.44	62.11 ± 4.55ᵃᵈ	77.87 ± 7.55	47.89 ± 5.22ᵃᵈ	187.12 ± 25.37	279.54 ± 39.56ᵃᵈ	54.87 ± 5.22	40.82 ± 4.33ᵃᵈ
Group D	37	29.85 ± 3.32	71.34 ± 6.67ᵃᵇᶜᵈ	78.23 ± 8.85	41.23 ± 4.43ᵃᵇᶜᵈ	186.89 ± 24.55	318.89 ± 51.22ᵃᵇᶜᵈ	55.67 ± 6.23	32.19 ± 3.65ᵃᵇᶜᵈ
*F*-value		0.094	129.329	0.183	43.483	0.014	21.983	0.262	70.613
*p*-value		0.963	<0.001	0.908	<0.001	0.998	<0.001	0.853	<0.001

Data are presented as mean ± SD. 5-HT, 5-hydroxytryptamine; CGRP, calcitonin gene-related peptide; GABA, *γ*-aminobutyric acid; ACh, acetylcholine. ᵃ*p* < 0.05 vs. Group A; ᵇ*p* < 0.05 vs. Group B; ᶜ*p* < 0.05 vs. Group C; ᵈ*p* < 0.05 vs. baseline within the same group.

#### Serotonin and inhibitory neurotransmitter levels

Serum 5-HT concentrations increased significantly in all treatment groups, with the combined therapy producing the most pronounced elevation. From similar baseline values of approximately 30 ng/mL across groups, Group D achieved post-treatment 5-HT levels of 71.34 ± 6.67 ng/mL (*p* < 0.05), significantly higher than Groups C (62.11 ± 4.55 ng/mL), B (61.89 ± 4.62 ng/mL), and A (48.89 ± 3.31 ng/mL) (*F* = 129.329, *p* < 0.001). GABA levels followed a similar trajectory, with Group D demonstrating the greatest increase to 318.89 ± 51.22 nmol/L (*p* < 0.05), significantly exceeding post-treatment values in Groups C (279.54 ± 39.56 nmol/L), B (278.23 ± 35.43 nmol/L), and A (244.55 ± 27.88 nmol/L) (*F* = 21.983, *p* < 0.001). Both individual therapy groups showed significantly higher post-treatment 5-HT and GABA levels compared to routine intervention (all *p* < 0.05).

#### Calcitonin gene-related peptide and acetylcholine levels

Conversely, serum CGRP levels decreased significantly following treatment across all groups. Group D exhibited the most substantial reduction, with levels decreasing from 78.23 ± 8.85 ng/L at baseline to 41.23 ± 4.43 ng/L after treatment (*p* < 0.05). This post-treatment CGRP concentration was significantly lower than those in Groups C (47.89 ± 5.22 ng/L), B (48.01 ± 5.48 ng/L), and A (55.47 ± 6.19 ng/L) (*F* = 43.483, *p* < 0.001). ACh levels demonstrated parallel reductions, with Group D achieving the lowest post-treatment concentration of 32.19 ± 3.65 nmol/L (*p* < 0.05) compared to Groups C (40.82 ± 4.33 nmol/L), B (41.15 ± 5.01 nmol/L), and A (46.87 ± 4.43 nmol/L) (*F* = 70.613, *p* < 0.001). For both CGRP and ACh, individual therapy groups showed significantly greater reductions compared to routine intervention (all *p* < 0.05).

## Discussion

The present study demonstrated that biofeedback combined with vestibular rehabilitation therapy produced superior clinical outcomes compared to either therapy alone or routine intervention in patients with VM. The combined therapy group achieved a total effective rate of 94.59%, significantly exceeding those of the individual therapy and routine intervention groups. Furthermore, patients receiving combined therapy exhibited greater improvements in anxiety and depression symptoms, vertigo disability, balance function, vestibular function parameters, cerebral blood flow, and serum biomarker profiles.

VM presents with complex and diverse clinical manifestations, including visually induced vertigo, positional vertigo, spontaneous vertigo, and movement-provoked dizziness with imbalance. These symptoms substantially impair patients’ quality of life, work productivity, and mental health, while imposing considerable socioeconomic burden. Although routine intervention provides some degree of symptom control, treatment outcomes often remain suboptimal (22), highlighting the need for more effective therapeutic strategies.

Our findings revealed that patients in the combined therapy group demonstrated significantly lower HADS and DHI scores and higher BBS scores compared to those receiving individual therapies or routine intervention. Anxiety and depression were assessed because they are highly comorbid with VM, acting as both consequences of recurrent vertigo and amplifiers of symptom perception, and because biofeedback acts directly on autonomic and affective regulation; the HADS therefore offered a direct readout of this pathway. These results suggest that the combined approach more effectively alleviates psychological distress and vertigo-related disability while enhancing functional balance. The observed improvements in vestibular function parameters, including spontaneous nystagmus velocity, canal paresis, and directional preponderance, further support the clinical efficacy of combined therapy. Although no single vestibular parameter is pathognomonic for VM, spontaneous nystagmus, canal paresis, and directional preponderance were examined together because they provide objective, examiner-independent indices of central vestibular compensation that corroborate the patient-reported DHI, allowing the functional gains of rehabilitation to be quantified rather than merely inferred.

The superior outcomes associated with combined therapy may be attributed to complementary mechanisms of action. Biofeedback therapy can reduce sympathetic nervous system overactivity and decrease central nervous system sensitization by training patients to consciously regulate their physiological state, thereby helping to alleviate anxiety and depression (15). Vestibular rehabilitation directly promotes vestibular-visual-proprioceptive integration through targeted exercises, strengthening vestibular compensation and improving balance function and vestibular parameters (23). When applied in combination, the autonomic stabilization achieved through biofeedback may enhance patient tolerance and compliance during vestibular rehabilitation, enabling the brain to more efficiently process sensory conflict signals generated during training. This synergistic interaction likely accelerates the vestibular compensation process and contributes to the enhanced clinical efficacy observed in the combined therapy group.

The present study also examined treatment effects on serum biomarkers implicated in VM pathophysiology. Our results showed that combined therapy produced the most favorable biomarker modulation, characterized by increased 5-HT and GABA levels and decreased CGRP and ACh levels compared to other treatment groups.

The observed increase in serum 5-HT levels following combined therapy is clinically relevant, as serotonergic dysfunction has been implicated in both migraine and vestibular disorders (11). Enhanced 5-HT signaling may contribute to improved vestibular system stability and reduced headache frequency (24). Similarly, the elevation in GABA levels suggests strengthened inhibitory neurotransmission, which is essential for maintaining central vestibular stability and may contribute to reduced symptom severity (25).

Conversely, the reduction in serum CGRP levels following treatment is noteworthy given the established role of CGRP in migraine pathophysiology. CGRP and its receptors are widely distributed throughout the vestibular system, and elevated CGRP levels have been associated with both headache and vertigo symptoms (26, 27). The decrease in CGRP observed in our study, particularly in the combined therapy group, suggests that biofeedback-induced relaxation may inhibit trigeminal vascular system activation, thereby reducing CGRP release. This reduction may normalize cerebrovascular tone, as reflected by the decreased cerebral blood flow velocities observed across all arterial territories.

The reduction in ACh levels following treatment may reflect decreased cholinergic excitatory drive within the vestibular system. Combined therapy appeared to synergistically reduce both CGRP and ACh levels, potentially stabilizing vestibular nucleus activity and contributing to symptom improvement.

Transcranial Doppler assessment revealed significant reductions in mean blood flow velocities in the anterior, middle, and posterior cerebral arteries following treatment, with the combined therapy group showing the greatest improvements. Cerebral blood flow was included as an outcome because trigemino-vascular activation and altered cerebrovascular tone are implicated in migraine pathophysiology, and transcranial Doppler provided a non-invasive means of testing whether the hypothesized reduction in CGRP-mediated vasoactivity was accompanied by measurable hemodynamic change. Elevated cerebral blood flow velocities at baseline may reflect cerebrovascular dysregulation associated with VM. The normalization of these parameters following treatment, particularly with combined therapy, suggests improved cerebrovascular homeostasis. This improvement may be mechanistically linked to reduced CGRP-mediated vasodilation and decreased sympathetic nervous system activity achieved through biofeedback training (28, 29).

**Figure 4:**
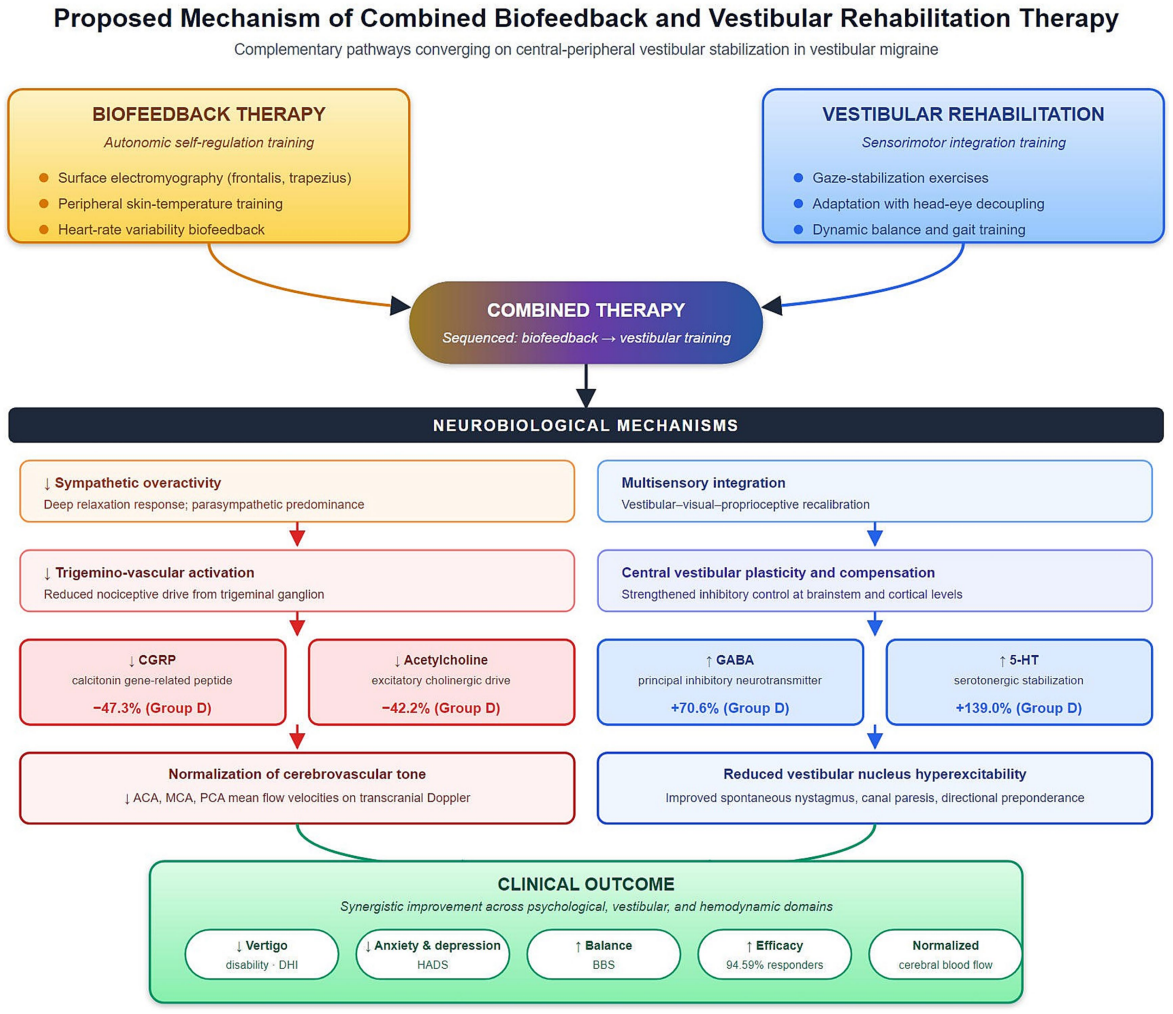
Proposed mechanism of combined biofeedback and vestibular rehabilitation therapy in vestibular migraine. Schematic representation of the two complementary pathways activated by the combined intervention. Biofeedback therapy acts primarily through autonomic self-regulation, attenuating sympathetic overactivity and trigemino-vascular activation, thereby lowering serum CGRP and acetylcholine (ACh) levels and normalizing cerebrovascular tone (left, red pathway). Vestibular rehabilitation promotes multisensory integration and central vestibular plasticity, strengthening inhibitory control and elevating serum *γ*-aminobutyric acid (GABA) and 5-hydroxytryptamine (5-HT) levels (right, blue pathway). Convergence of these pathways produces synergistic improvement in vertigo, anxiety and depression, balance function, cerebral blood flow, and overall treatment efficacy. Percent-change values are those observed in Group D.

Based on our findings, we propose that combined biofeedback and vestibular rehabilitation therapy may exert therapeutic effects through multiple complementary mechanisms. Biofeedback training induces a deep relaxation state that inhibits sympathetic overactivation and trigeminal vascular system excitation, reducing CGRP release and normalizing cerebrovascular tone. The relaxation response is closely associated with GABAergic system activation, while regulation of the limbic system and brainstem may enhance serotonergic neuron function, promoting increased 5-HT levels. Concurrently, vestibular rehabilitation provides structured sensorimotor integration training that promotes central nervous system plasticity and strengthens inhibitory control. The synergistic effects of these two interventions may more comprehensively address the multifaceted pathophysiology of VM than either approach alone. A schematic representation of these proposed complementary pathways and their convergence on clinical outcomes is provided in Figure 4.

Several limitations of this study should be acknowledged. First, the non-randomized study design may introduce selection bias, although baseline characteristics were comparable across groups. Second, the sample size of 148 patients from a single center limits the generalizability of our findings. Third, the 4-week follow-up period does not allow assessment of long-term treatment durability. We regard this as the most important constraint on the present findings. The 4-week window was chosen to capture the immediate physiological and biochemical response to a defined treatment course and to align outcome assessment with the period of supervised intervention, but it cannot establish whether the observed gains in symptom control, vestibular function, and biomarker profiles are sustained once active therapy ends. VM characteristically follows a relapsing–remitting course, and meaningful conclusions about durability, relapse rates, and the need for maintenance or booster sessions will require substantially longer observation. The benefits reported here should therefore be interpreted as short-term effects, and we explicitly caution against extrapolating them to durable, long-term efficacy until confirmed by studies with extended follow-up of at least 3 to 6 months. Fourth, outcome assessors were blinded to group allocation, but patients and treating therapists could not be blinded to treatment assignment, which may introduce performance bias. Finally, serum biomarker levels may not directly reflect central nervous system neurotransmitter concentrations.

Future research should address these limitations through multicenter randomized controlled trials with larger sample sizes and longer follow-up periods. Investigation of additional biomarkers and neuroimaging correlates may further elucidate the mechanisms underlying combined therapy efficacy. Studies comparing different treatment sequencing and dosing regimens may help optimize the combined therapy protocol for clinical implementation.

## Conclusion

In conclusion, biofeedback combined with vestibular rehabilitation therapy demonstrated superior efficacy compared to either therapy alone or routine intervention in patients with VM. This combined approach effectively regulated serum levels of 5-HT, CGRP, GABA, and ACh, reduced anxiety, depression, and vertigo disability, and improved balance function, vestibular function, and cerebral blood flow. These findings support the clinical application of combined biofeedback and vestibular rehabilitation as a promising non-pharmacological treatment strategy for VM.

## Data Availability

The raw data supporting the conclusions of this article will be made available by the authors, without undue reservation.
